# Dataset of normalized probability distributions of virtual bond lengths, bond angles, and dihedral angles for the coarse-grained single-stranded DNA structures

**DOI:** 10.1016/j.dib.2022.108284

**Published:** 2022-05-15

**Authors:** Jun-Lin Qian, Li-Zhen Sun

**Affiliations:** Department of Applied Physics, Zhejiang University of Technology, China

**Keywords:** Unstructured ssDNA, Conformational change, Coarse-grained potential, Computational simulation

## Abstract

The utility of the coarse-grained (CG) single-stranded DNA (ssDNA) model can drastically reduce the compute time for simulating the ssDNA dynamics. The model-matched CG potentials and the inherent potential constants can be derived by coarse-graining the experimentally measured ssDNA structures. A useful and widespread treatment of the CG model is to use three different pseudo-atoms P, S, and B to represent the atomic groups of phosphate, sugar, and base, respectively, in each nucleotide of the ssDNA structures. The three pseudo-atoms generate nine types of the structural parameters to characterize the unstructured ssDNA conformations, including three (virtual) bond lengths (P-S, S-B, and S-P) between two neighbouring beads, four bond angles (P-S-P, S-P-S, P-S-B, and B-S-P) between three adjacent bonds, and two dihedral angles (P-S-P-S and S-P-S-P) between three successive bonds. This paper mainly presents the data of normalized probability distributions of the bond lengths, bond angles, and dihedral angles for the CG ssDNAs.

## Specifications Table

**Table:** 

Subject	Biophysics
Specific subject area	The coarse-grained model for simulating the single-stranded DNA dynamics
Type of data	Table and figure
How the data were acquired	The normalized probabilities of the structural parameters are statistically obtained by coarse-graining the experimentally detected ssDNA structures [1], [2], [3]. A software UCSF Chimera (version 1.11.2) [4] is used to delete the unnecessary atoms in the all-atom structures. A software X3DNA (version 2.4) [5] is used to label the unpaired nucleotides.
Data format	Raw and analysed
Description of data collection	The normalized probabilities are calculated as following: (1) For each nucleotide, we calculate the centers of mass of the phosphate (P), sugar (S), and base thymine (B(T)). (2) For each ssDNA chain, the structural parameters then can be calculated, including • virtual bond lengths P*_i_*-S*_i_*, S*_i_*-B*_i_*(T), and S*_i_*-P*_i_*_+1_; • virtual bond angles P*_i_*-S*_i_*-P*_i_*_+1_, S*_i_*-P*_i+_*_1_-S*_i+_*_1_, P*_i_*-S*_i_*-B*_i_*(T), and B*_i_*(T)-S*_i_*-P*_i_*_+1_; • and dihedral angles P*_i_*-S*_i_*-P*_i_*_+1_-S*_i_*_+1_ and S*_i_*_-1_-P*_i_*-S*_i_*-P*_i_*_+1_; where the subscript represents the nucleotide index. and (3) the normalized probabilities of the structural parameters can be statistically analyzed from the corresponding parameters obtained in the step above.
Data source location	All selected 3D ssDNA structures are downloaded from the website of the protein data bank [6] (access to https://www.rcsb.org/).
Data accessibility	Repository name: Mendeley Data Data identification number: doi:10.17632/nbd83424kc.2 Direct link to the dataset: https://data.mendeley.com/datasets/nbd83424kc/2
Related research article	L.Z Sun, J.L. Qian, P. Cai, H.X. Hu, X. Xu, M.B. Luo, Mg^2+^ effects on the single-stranded DNA conformations and nanopore translocation dynamics, Polymer, 250 (2022) 124895

## Value of the Data

• These data can provide an insightful view of the conformational changes for the unstructured ssDNAs.
• These data can be used to support the developments of new CG ssDNA models or the modifications of the existing models.
• The researchers who are interested in the ssDNA dynamics and simulation models will benefit from the data presented in this paper.

## 1 Data Description

**Fig. 1:**
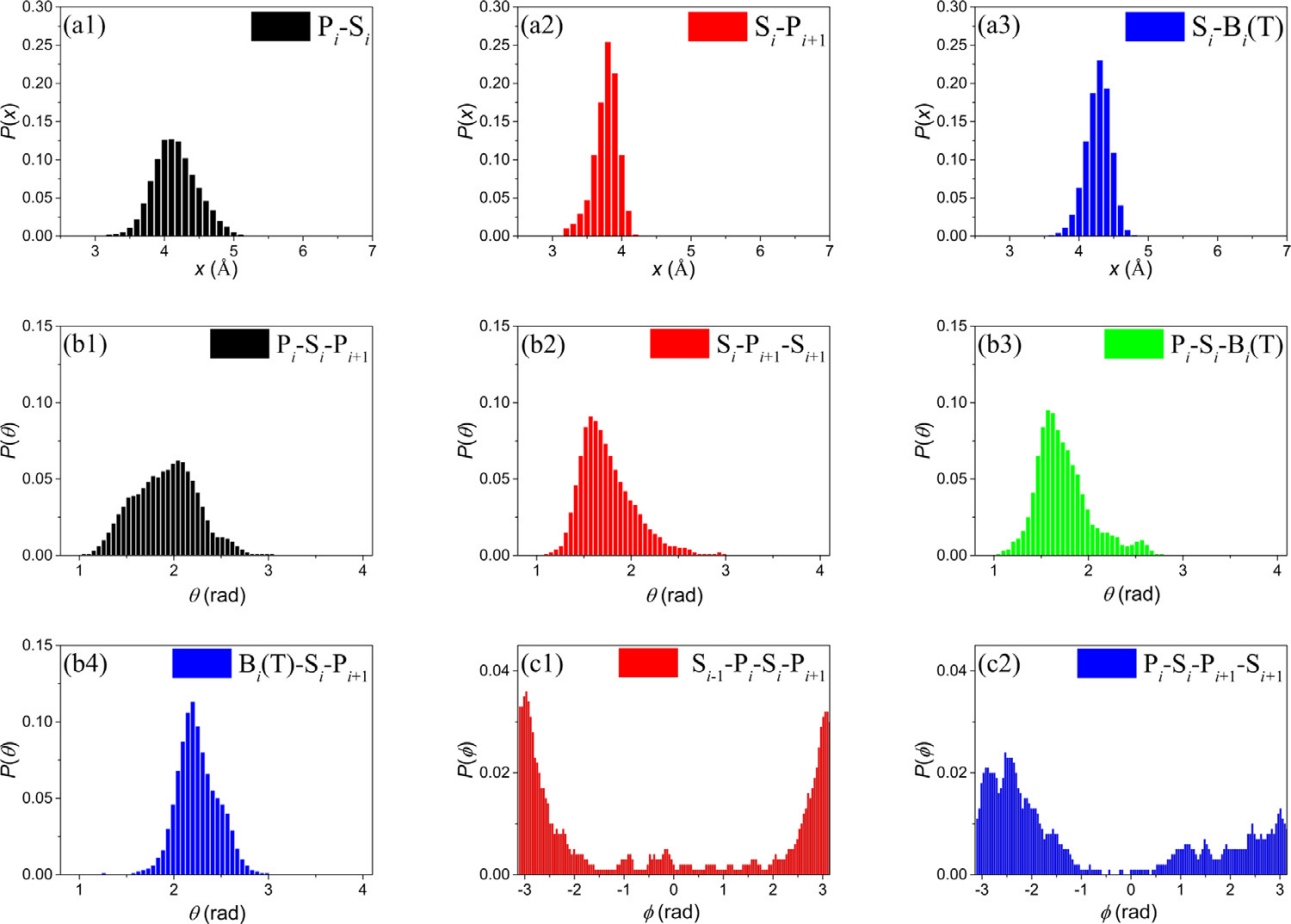
(a1)–(a3) show the normalized probability distributions of the virtual bond lengths P*_i_*-S*_i_*, S*_i_*-P*_i_*_+1_, and S*_i_*-B*_i_*(T), respectively. (b1)–(b4) show the normalized probability distributions of bond angles P*_i_*-S*_i_*-P*_i_*_+1_, S*_i_*-P*_i+_*_1_-S*_i+_*_1_, P*_i_*-S*_i_*-B*_i_*(T), and B*_i_*(T)-S*_i_*-P*_i_*_+1_, respectively. (c1) and (c2) show the normalized probability distributions of the dihedral angles S*_i_*_-1_-P*_i_*-S*_i_*-P*_i_*_+1_ and P*_i_*-S*_i_*-P*_i_*_+1_-S*_i_*_+1_, respectively. Here the subscript represents the nucleotide index.

The PDB identifications (PDBids) and the basic information (including the number of chains *N*_c_, the number of all nucleotides *N*_n_, and the ssDNA types) of the selected ssDNAs are summarized in Table 1. More detailed information such as the sequences are deposited in the Mendeley Data database (Table 1: sequences of the selected ssDNA structures). Fig. 1 shows the normalized probability distributions of structural information the virtual bond lengths (Fig. 1(a1)–(a3)), the bond angles (Fig. 1(b1)–(b4)), and dihedral angles (Fig. 1(c1) and (c2)) for the CG ssDNAs. The ssDNA backbone-involved structural parameters, such as the bond length P-S, the bond angle P-S-P, and the dihedral angle P-S-P-S, are calculated from all selected ssDNA structures. As we mainly focus on the ssDNA polythymine poly(T), the base-involved structural parameters such as the bond length S-B and bond angle P-S-B, are obtained from the thymine-involved structures. In addition, the base-involved dihedral angles are not calculated as their effects on the conformations of the unstructured polythymine are weak [2]. The data are also deposited in the Mendeley Data database (Tables 2–4).

**Table 1:** The basic information of the selected ssDNA structures.

PDBid	*N*_c_^a^	*N*_n_^b^	type^c^	PDBid	*N*_c_	*N*_n_	type
1eyg	2	70	poly(T)	4jlj	2	34	mixed
1jmc	1	8	poly(C)	4jrq	2	26	poly(A)
1pa6	3	36	mixed	4js4	2	32	poly(A)
1ph1	3	39	mixed	4js5	2	26	poly(T)
1ph2	3	35	mixed	4kdp	2	34	mixed
1ph3	3	38	mixed	4ki2	4	44	mixed
1ph4	3	38	mixed	4noe	1	30	mixed
1ph5	3	36	mixed	4ou6	1	10	poly(T)
1ph6	3	37	mixed	4ou7	1	10	poly(T)
1ph7	3	36	mixed	4owx	1	12	poly(T)
1ph8	3	38	mixed	4pog	4	120	poly(T)
1ph9	3	38	mixed	4pso	4	40	poly(T)
1phj	3	37	mixed	4qgu	2	24	mixed
1s40	1	11	mixed	5cd4	2	120	mixed
2c62	1	20	mixed	5eax	2	34	poly(T)
2ccz	1	15	poly(T)	5fhd	2	38	mixed
2vw9	1	35	poly(T)	5h1b	1	9	poly(T)
3a5u	1	31	poly(C)	5odl	1	9	poly(T)
3cmu	1	18	poly(T)	5orq	1	10	mixed
3cmw	2	30	poly(T)	5u8t	1	14	poly(T)
3ugo	2	22	mixed	5usb	1	9	mixed
3ugp	2	22	mixed	5usn	1	9	mixed
3ulp	2	70	poly(T)	5uso	1	9	mixed
3vdy	5	175	poly(T)	5xrz	1	40	mixed
4bhm	3	24	poly(T)	5xs0	3	27	poly(C)
4g0r	1	10	mixed	5zg9	1	20	poly(T)
4gnx	2	124	poly(T)	5zva	1	10	mixed
4gop	2	64	poly(T)	5zvb	1	9	mixed
4hid	1	9	mixed	6fwr	1	11	poly(T)
4hik	1	9	mixed	6fws	2	21	poly(T)
4him	1	9	mixed	6i52	1	20	poly(T)
4hj5	1	9	mixed	6irq	2	50	poly(T)
4hj7	1	9	mixed	6jdg	3	60	poly(T)
4hj8	1	9	mixed	6kbs	1	10	mixed
4hj9	1	10	mixed	6pij	3	96	mixed
4hja	1	11	mixed	6qem	1	36	poly(T)

^a^*N*_c_ denotes the number of chains in a structural file. ^b^*N*_n_ denotes total number of nucleotides in a structural file. ^c^The type named “mixed” represents the corresponding ssDNA with different compositions.

## 2 Experimental Design, Materials and Methods

The statistical analysis of the structural information for the ssDNAs mainly involves three steps: (1) selecting appropriate all-atom ssDNA structures; (2) coarse-graining the selected structures; and (3) calculating the CG structural parameters. The details are described as following:

*Selection of the all-atom ssDNA structures.* The experimentally measured all-atom ssDNA structures are download from the protein data bank [6]. The selection of the structures based on the following criteria: (1) the ssDNA is bound to proteins to avoid the formation of helical structures [7]; and (2) there are at least 8 consecutive unpaired nucleotides. A total of 72 ssDNA structure files with PDB format (see the PDBids in Table I) are used. Then we use the visualization tool UCSF Chimera [4] to delete unnecessary molecules (such as proteins and waters), ions and hydrogen atoms of the ssDNAs, and use the software package X3DNA [5] to label the unpaired nucleotides.

*CG structures of the ssDNAs.* We calculate the centers of mass for the atomic groups of the phosphate (P), sugar (S), and base (B) in each unpaired nucleotide. In particular, for the nucleotide *i*, the phosphate includes the atom phosphor and the directly bonded oxygen atoms (here an oxygen named O3’ in PDB files in fact belongs to the nucleotide *i*-1), the sugar group includes the sugar ring and an atom named C5’, and the base group includes other atoms in this nucleotide except the atom O3’ as it belongs to the phosphate of nucleotide *i*+1. In the CG structures, the atomic groups are represented by the three types of pseudo-atoms P, S, and B located the corresponding centers of mass. The pseudo-atoms are assumed to be connected by virtual bonds.

*Calculation of the structural parameters.* For all CG ssDNA structures, we calculate the virtual bond lengths between two neighbouring pseudo-atoms (including P-S, S-P, and S-B(T)), the bond angles between two adjacent virtual bonds (including P-S-P, S-P-S, P-S-B(T), and B(T)-S-P), and the dihedral angles formed by three successive bonds (including P-S-P-S, S-P-S-P) in the backbone. Based on the calculation results, the normalized probability distribution for the corresponding structural parameters then can be statistically analyzed (see Fig. 1).

## CRediT authorship contribution statement

**Jun-Lin Qian:** Resources, Investigation, Visualization, Writing – original draft. **Li-Zhen Sun:** Conceptualization, Methodology, Software, Writing – review & editing.

## Declaration of Competing Interest

The authors declare that they have no known competing financial interests or personal relationships that could have appeared to influence the work reported in this paper.

## Data Availability

Normalized probabilities of structural parameters for CG ssDNAs (Original data) (Mendeley Data).
